# Factors that influence access to mental health services in South-Eastern Europe

**DOI:** 10.1186/s13033-018-0255-6

**Published:** 2018-12-07

**Authors:** Andreea Raluca Tirintica, Ivana Andjelkovic, Orela Sota, Mihail Cristian Pirlog, Maria Stoyanova, Adriana Mihai, Neal Wallace

**Affiliations:** 1 University of Medicine and Pharmacy Targu Mures, Târgu Mureş, Romania; 2 NGO Indigo in Presevo Refugee Reception Center, Preševo, Serbia; 3 Health Center No. 3, Fier, Albania; 4 University of Medicine and Pharmacy Craiova, Craiova, Romania; 5 Mental Health Center “Prof. Nikola Shipkovenski”, Sofia, Bulgaria; 6 IPPD Institute of Psychotherapy and Personal Development, Târgu Mureş, Romania; 7 OHSU-PSU School of Public Health, Portland, OR USA

**Keywords:** Access, Mental health services, Factors, Europe

## Abstract

**Introduction:**

Access to mental health (MH) services is unequal worldwide and changes are required in this respect.

**Objectives:**

Our aim was to identify the delay to the first psychiatry consult and to understand patients’ characteristics and perspectives on the factors that may influence the delay, among a sample of participants from three Southeastern European Countries.

**Materials and methods:**

The WHO Pathway Encounter Form questionnaire was applied in 400 patients “new cases” and a questionnaire on the factors influencing the access was administered to the same patients, as well as to their caretakers and MH providers.

**Result and discussions:**

The average profile of the patient “new case” was: married female older than 40 years, with an average economic status and no MH history. The mean delay was up to 3 months and the most important factors that were influencing the delay were stigma and lack of knowledge regarding MH problems and available current treatments.

**Conclusions:**

Future policies trying to improve the access to psychiatric care should focus on increasing awareness about MH problems in the general population.

## Introduction

In many parts of the world, patients’ access to mental health (MH) services is unequal and would require changes in this respect [1]. Studies in this field show that although it seems that Eastern Europe countries are aligned to international statistics regarding the delay to the first psychiatry consult, this duration varies greatly from one patient to another [1, 2].

The results of a large national survey in Germany among patients with schizophrenia and comorbid depression reveal that in general people believe that psychiatrists can only treat schizophrenia, not depression, because schizophrenia has biological uncontrollable causes, while depression is a consequence of the failure to adapt to social life [3]. Consequently, people with depression rather seek help from friends or colleagues. A study in Central Haiti reveals a high mental health burden of Major depressive episode (MDE) and Posttraumatic stress disorder (PTSD) among school-going youth, where 88.6% had accessed MH services in the health sector [4].

A multicenter study which examined the pathways to MH care in Italy underlines the necessity of a stronger collaboration of psychiatrists with general practitioners (GPs), hospital doctors and psychologists, because GPs are the main referral points for mental health care and this could improve the access to care [5, 6]. It seems that a negative perception of MH problems and treatments impedes the access to care [7].

A national survey in the U.S. shows that only 1 in 3 patients with mental illness seek medical help. Wittchen et al. show that mental illness affects 27% of Europe’s population, but 74% of them do not receive treatment and that there is no single factor to explain this [8]. Most evidence suggests stigma and distrust to psychiatric treatment as the most important factors influencing the delay [9]. Studies suggest that stigma-related MH knowledge is associated with attitudes towards MH problems, so an intervention in this area could improve the access [10]. Internalized Sigma of Mental Illness (ISMI) is correlated with higher depression, lower self-esteem, and higher symptom severity [11] and initial evidence shows that stigma could be a factor contributing to suicidality [12].

UK and U.S. studies show that there are significant barriers that increase the impact of mental illness: socio-cultural (religious beliefs and practices), systemic (problem in the organization of MH services), economic (health insurance) and individual (denial of psychological problems). In the United States, patients are more likely to seek psychiatric help if the family is supportive and a family member had a positive experience with MH services [9, 13]. Another study conducted in the United Arab Emirates and Jordan among Muslim female students show that they prefer to use prayers and traditional medicines than to seek help from MH professionals [14]. In the United States, personal beliefs (fatalism) were significantly associated with a low use of psychiatric care among Latinos [15].

Moreover, there are some believes which reduce the likelihood that patients seek psychiatric help such as ineffectiveness of psychiatric treatments [16], fear of being stigmatized because of having a mental illness and believes that one must resolve its own problems [17].

Some patients avoid psychiatric services because they fear ECT [18]. It seems that our society still show the old-fashioned ideas and ignorance regarding mental illness and specialized treatment.

Studies focused on this topic are almost missing in the Southeastern European countries (e.g. [2] and the studies which have been done referred to the duration to treatment of first time admission patients and there are no studies focused on the reason for the delay of the first psychiatric consult.

## Objectives

Our aims were to identify the delay to the first psychiatry consult and to understand the patient’s characteristics and perspectives on the factors that may influence this delay among a sample of patients from Southeastern European Countries.

## Materials and methods

### Design overview

The study was designed with the aim to determine which factors may be correlated to the duration of the delay in contacting MH services by individuals who encounter MH problems.

We conducted the study in three South-Eastern European countries: Romania (two sites), Bulgaria and Albania. In each country the sample included the patients, caregivers and MH professionals within each site. The sites where we conducted the study were psychiatry clinics in Romania and general health centers in Bulgaria and Albania. The sites were representative of their respective countries clinics (typical and important cities in each country) and they were selected because they were accessible to researchers.

The design of the study was correlational. We compared patients’ characteristics and a number of different external factors in order to understand from whom and why delays to treatment may be occurring.

### Sampling and data collection

The study included three different samples: the patients, their caregivers and their MH providers. The patients were chosen if they fulfilled the following criteria: having not been admitted to the psychiatric institutions in the last 2 years; being older than 18; being able to understand the content of the questionnaire (we excluded patients with delirium or significant cognitive impairment). We enrolled patients in the waiting rooms of the clinics.

### The caregiver and MH professional samples were based on the individual patients enrolled in the study

The caregivers were defined as persons who took care of the patients who took part in our study (mostly close family members) who were identified at same time as patient, in the waiting rooms of the clinics.

MH professionals were defined as the professionals (psychiatrists, psychologists, neuropsychiatrists, clinical social workers etc.) who provided services devoted to the treatment of mental health problems to the enrolled patients. We sought prior permission from each clinic to match enrolled patients with their clinic providers and then asked them to participate to the survey.

The estimated subject population included a total of 400 participants: 100 patients “new cases” (who have not been admitted in a psychiatry clinic in the last 2 years) for each site. They were interviewed in the period of May–August 2015. All participants signed an informed consent. The study was approved by the national ethic committees.

We used two questionnaires: A modified version of WHO Pathway Encounter Form questionnaire was applied to each patient, in order to determine the delay to the first psychiatry consult. This questionnaire collects information about demographics, the first decision to ask for help, symptoms and other diagnostics, the delay to the first consultation. In the second questionnaire participants had to rank 6 categories of factors (stigma—against psychiatry or mental disorders, feelings of the patient, lack of knowledge—regarding mental disorders and available treatments, availability of MHS, personal beliefs of the patient, social support) according to their importance for the subject. This questionnaire was applied to the patients, caretakers and MH providers, in order to assess the factors influencing the patient from three perspectives. The average duration of the interview was around 15 min for each participant. In the present study we have focused only on the patients’ sample. A comparative study between the three samples will be reported further.

### Measures

In the strict sense, there were no dependent and independent variables in our study, because it was a correlational one. However, we made a provisional division and defined as the main dependent variable the duration of the delay measured in weeks between the first appearance of symptoms and the first contact with MH services. We defined as independent separate variables the factors (stigma, feelings, lack of knowledge, availability of MH services, personal beliefs and social support) measured on a scale from 1 to 6, according to its importance for the delay in contacting MH services; the factor ranked as the most important form the six categories and the demographic variables (sex, age, marital status, economic status, existence of previous MH history. Other patients variable which were included in the analyses were who suggested the consultation (patient, family or doctor), with whom the first consultation happened (GP, psychiatrist, neurologist, etc.) and diagnosis.

### Analytic methods

First, we estimated the mean and median values of the delay variable and mean ranking scores of the factor variables. The patients’ responses form the three sites were pooled together. Furthermore, correlation between different patient characteristics and duration of delay was assessed through analysis of variance. Finally correlation between factors’ choice and duration of the delay was examined using analysis of variance.

ANOVA was used to examine the relationship between factors and the duration of the delay. The dependent variable was duration of the first delay (between the first emergence of symptoms and the first contact with MH system) and the six categories of factors ranked by the participants were the independent variable.

## Results

We evaluated 400 patients. The main characteristics of the patients were: female (67.3%), married (61.3%), older than 40 years, with average economic status—(77%), without mental health history (62%) and more than 90% lived in the research area (Table 1).

**Table 1:** Patient socio-demographics by time to the first MH visit

Measure	Total	Duration to the first MH visit			P < *
		Less than 3 months	3 months to 1 year	More than 1 year	
Sample					
N	400	205	70	88	
%	100%	51.3%	17.5%	22%	
Gender					0.577
Female	67.3%	69.8%	55.7%	72.7%	
Male	32.8%	30.2%	44.3%	27.3%	
Age					0.516
18–30	14.3%	12.7%	15.7%	9.1%	
31–40	15.3%	13.7%	28.6%	6.8%	
41–50	22.3%	20.5%	24.3%	21.6%	
51–60	34.3%	36.1%	20.0%	53.4%	
> 60	14%	17.1%	11.4%	9.1%	
Marital status					0.683
Single	22.1%	18.5%	28.6%	17.0%	
Married	61.4%	62.4%	58.6%	64.8%	
Other	16.5%	19.0%	12.9%	18.2%	
Economic status					0.240
High	4.8%	3.9%	5.7%	4.5%	
Average	77%	72.2%	80.0%	80.7%	
Poor	18.2%	23.9%	14.3%	14.8%	
Who suggested consultation					0.008
Patient	39.3%	44.4%	28.6%	44.3%	
Family	49.3%	44.9%	60.0%	38.6%	
Doctor	11.4%	10.7%	11.4%	17.0%	
Diagnosis					0.000
Mood disorder	51.3%	50.2%	47.1%	60.2%	
Neurotic disorder	19%	20.5%	24.3%	5.7%	
Psychotic disorder	12%	11.7%	11.4%	12.5%	
Alcohol dependency	7%	2.9%	7.1%	19.3%	
Organic mental disorder	5.7%	9.8%	1.4%	2.3%	
Other mental disorder	5%	4.9%	8.6%	0.00%	

* < 0.05 for differences by duration of the delay to the first visit

The first psychiatric consult was asked predominantly by the family in Albania (84%) and Romania (50%). In Bulgaria, patients decided from themselves in 41% of cases, 32% took advice from the family and 27% were suggested by the doctor. Almost half of the new cases (49.8%) visited psychiatrists directly, with GPs being the close second (44%).

The duration of the delay was correlated with who suggested the first psychiatric consult and with the category of diagnosis. The patients to whom a doctor suggested a consult to MH provider had the longest delays (111.27 weeks as mean value and 14 weeks as median). The patients who decided themselves to visit a psychiatrist had the shortest delay (the mean value was 40.82 and the median was 8 weeks). The patients with alcohol dependency had the longest delays according to category of diagnosis (170.96 as mean value and 103.5 weeks as median). Almost two-thirds—60.7% of them had delays longer than 1 year. Psychotic patients also had a higher mean value (82.15 weeks). The patients with neurotic symptoms had the shortest delays (23.55 weeks as mean and 8 weeks as median value).

**Fig. 1:**
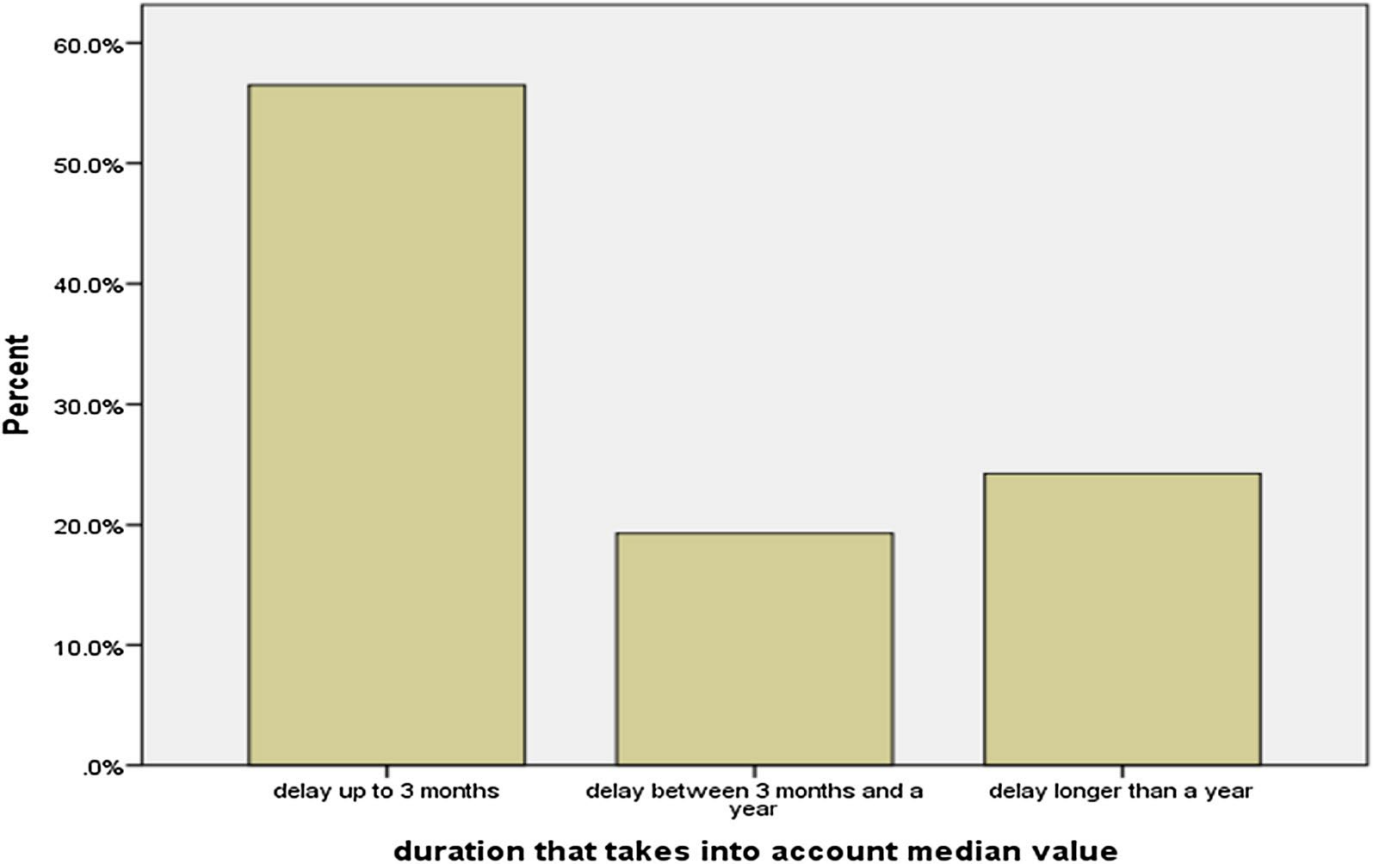
The duration of the delay to the first psychiatric consult

The average length of delay for the first psychiatric consult was 56.4 weeks (around 1 year), but this number was highly varying, as the standard deviation was: SD = 135.9 (the maximum being 1086 weeks, which is approximately 20.5 years). We calculated also the median value as a better approximation, which revealed that people usually wait 10 weeks before they seek for help for MH problems. The majority of patients had a delay of up to 3 months (Fig. 1). Still, a high percentage of patients had a delay that lasted more than a year and only 8% of them sought help during the first week of emergence of symptoms.

The factor ranked as the most important was Stigma, followed by Feelings and Lack of knowledge (Table 2).

Descriptive statistics showed differences in length of the delay between participants: those who chose Lack of Knowledge and Availability of MHS as the most important factors had longer delays (their median values were 12 weeks and respectively 16 weeks). Also, Stigma and Feelings were deemed more important and chosen more frequently than other factors. However, ANOVA analysis did not confirm a significant statistical difference.

However, when the delay was recoded from a dependent to a categorical variable (short delay was defined as “less than a week”, medium delay was between 1 week and 1 year and long delay was defined as “1 week to 1 year/more than a year”) and correlated to the variable factors, the correlation coefficient became statistically significant (Pearson Chi square = 22.766, df = 10, Sig. = 0.012). As Cramer’s V was 0.174 we can conclude that the strength of association was weak, but acceptable. Although Stigma was the most important factor for the delay in all of the three groups, Social support was important for those with short delays (18.8%), while Lack of knowledge was important for those with average and long delays (19.9% and respectively 30.7%).

Patients’ characteristics were assessed according to the duration of delay and its correlation with the factors (ex. Do patient’s characteristics variables that have differences in duration of the delay also show differences in factor choice?).

Surprisingly, the category of diagnosis was not correlated with the factors. However, our data showed that factors differ depending on who suggested the consultation (Table 3). Patients who entered MH system due to suggestion of other people (doctors and family) were more likely to choose Stigma as the most important factor (45.2% and respectively 39.1%), while patients who made that decision by themselves chosen Lack of knowledge as the most important (31.2%) and stigma as the second important factor (29.9%).

Another variable that was not correlated with the length of the delay but was correlated to the factor’s choice was the economic status. Participants with high economic status tended to choose stigma as the most important factor more often than those with average or poor status.

## Discussions

Patients’ characteristics reflect demographic data in epidemiological studies for psychiatric disorders. Women after 40 years seem to ask for help much easier than men and younger persons and the distance to MH services could be a determinant factor on the access to help.

The fact that the first psychiatric consult was asked predominantly by the family and the patients in all countries shows that—in order to improve the access to MH services—an important part could be promoting mental health in general population by helping them to understand how to recognize some psychiatric symptoms, when and where to ask for help and available treatments in our days. The understanding of MH disorders in general population could also contribute in diminishing stigma. Also, our results confirmed the importance of the GPs in the access to care and the impact that a good collaboration between GPs and other specialists could be beneficial for the patient.

Doctors’ suggestions to consult a MH provider are correlated with a longer delay. This could imply that other specialists are trying to manage psychiatric disorders for a while, avoiding addressing the patients to their psychiatrist colleagues. Those patients could have a lack of awareness for their own symptoms and therefore seek help only when they are officially given a referral by the doctor. The occurrences of a shorter delay in patients who ask for help by themselves prove the importance of awareness regarding MH symptoms and available treatments—for the individual. As expected, neurotic patients asked for professional help faster than other categories of patients. Patients with alcohol dependency had the longest delays, which suggest that this addiction is still stigmatized and not seen as a psychiatric problem—results which are similar with the literature.

The category of diagnosis also influenced the delay: the longest delay had the patients with alcohol dependency and the shortest—those with neurotic symptoms. Interestingly a long delay to the first psychiatric consult was observed also for psychotic patients—which suggest a lack of knowledge regarding psychiatric symptoms and ignorance towards the vulnerability of psychotic patients and the importance of early treatment. However, the category of diagnosis was not connected to choice of the factors (Table 1).

Almost a quarter of patients had a delay longer than a year. Long delays were associated with stigma and feelings of the patients, while short delays appeared when patients had good social supports. However, when family of doctors suggested the first psychiatric consult—patients tend to feel stigmatized and to have a longer delay. Those aspects suggest that the most important interventional projects should be based on increasing awareness towards MH problems and available treatments, in the general population. This could also diminish the stigma factor.

**Table 2:** First choice WHO patient access factors by duration to first MH visit. Stigma and lack of knowledge were more often chosen by patients with longer delays to the first psychiatric consult

Factors	Total	Duration to the first MH visit			P < *
		Less than 3 months	3 months to 1 year	More than 1 year	
					0.519
Stigma	38.5%	39.0%	*46.0%*	42.6%	
Feelings	21.8%	23.1%	17.8%	17.8%	
Lack of knowledge	21%	20.5%	14.9%	*23.9%*	
Availability	5.8%	6.5%	9.2%	5.1%	
Personal beliefs	7.3%	7.5%	8.6%	5.1%	
Social support	5.8%	3.4%	3.4%	*5.6%*	

* < 0.05 for differences by duration of the delay to the first visit

Italic values (46.0% and 23.9% and 5.6%) indicate differences in the delay to the first psychiatric visit. However, no significant statistical difference was confirmed by ANOVA

**Table 3:** First choice WHO patient access factors by selected demographics

Factors	% First choice WHO access factors						P < *
	Stigma (%)	Feelings (%)	Lack of knowledge (%)	Availability (%)	Personal beliefs (%)	Social support (%)	
Who suggested consultation							0.001
Patient	30.5	40.2	58.3	26.1	27.6	52.2	
Family	57.8	50.6	34.5	65.5	44.8	39.1	
Doctor	11.7	9.2	7.1	17.4	27.6	8.7	
Diagnosis							0.247
Mood disorder	46.8	51.7	64.3	43.5	41.4	52.2	
Neurotic disorder	18.8	23.0	14.3	26.1	13.8	21.7	
Psychotic disorder	13.6	11.5	6.0	13.0	20.7	13.0	
Alcohol dependency	10.4	0.00	6.0	8.7	13.8	4.3	
Organic mental disorder	5.8	5.7	6.0	4.3	10.3	0.00	
Other mental disorder	4.5	8.0	3.6	4.3	0.00	8.7	
Economic status							0.002
High	7.8	1.1	3.6	0.00	10.3	0.00	
Average	79.2	81.6	82.1	60.9	58.6	65.2	
Poor	13.0	17.2	14.3	39.1	31.0	34.8	

* < 0.05 for differences by factor’s choice (Chi square test)

The importance of stigma as a factor influencing the access to mental health care was underlined in many studies, and WHO’s Action Plan in Mental Health is trying to diminish stigma, in order to assure equal access to MH care all over the world [1]. The association between a short delay (less than a week) and social support also suggest the importance of increasing MH awareness in general population, as well as the association between lack of knowledge and long delays. However, more studies are needed in this direction, as our results did not confirm a significant statistical difference.

There was no correlation between categories of diagnosis and different factors, because patients consulting for psychotic or neurotic disorders had similar results. In the same time, it was very important who suggested the first consult, as the patients who entered MHS due to suggestions’ of family or other doctors felt that stigma was an important factor, while patients who decided themselves considered lack of knowledge the most important factor. Those results also support the benefits of MH support and information programs.

Different reasons could explain why the patients with high economic status tended to feel that they are more stigmatized than the patients with average or poor economic status.

## Limitations of the study

Our sampling is reasonably representative (not much limits on patient selection, clinics that are generally typical to countries), but not designed to be perfectly so (e.g. no random sampling of clinics based on geographic or other characteristics). Overall, our sampling reflects typical patients as seen in the study clinics over a 4 month period. While the clinics are generally representative of common MH providing organizations in each country, they are arguably a convenience sample (i.e. available to researchers) and may not fully represent the range of providers and their patients within each country.

One of the limitations of the study was that it was applied only in South-European countries—which have different health systems and social surroundings according to countries—therefore data may not be generalizable, but offer an important perspective of this topic in this specific region. Another limitation is that the sample was bigger in Romania, which could have impacted the results. As the samples were chosen for convenience, we do not know how representative they are.

The design of the research was correlational and since correlation doesn’t imply causation, we should be careful with the interpretation of the results: in order to draw causal conclusions, multi-variate testing should be done.

## Conclusions

WHO is constantly trying to improve the access to care. In this respect it is important to further investigate the delay to mental health care access. However, few studies were searching for the factors influencing the access to MH care and further research in this direction could be useful. Today’s health systems should be reformed in order to offer more information about available MHS (info at radio/TV, posters, booklets available at GP’s offices etc.). MH should be discussed in schools and media, so people have more information to recognize their problems. Stigma and different ways of fighting it should be discussed in events of psychiatrics’ associations, TV’s debates and articles in local newspapers, so that people to be able to share their experiences.

Our study showed that GPs are very important channel of communication between patients and health systems, so they should have more training about recognizing MH problems, instead of trying to treat them.

Future policies should focus on increasing awareness (in general population) towards MH problems and available treatments, in order to diminish stigma and assure a better access to care.

## Abbreviations

MH: mental health
WHO: World Health Organization
GP: general practitioner
MDE: major depressive episode
PTSD: posttraumatic stress disorder
ISMI: Internalized Stigma of Mental Illness
